# Generation of somatic *de novo* structural variation as a hallmark of cellular senescence in human lung fibroblasts

**DOI:** 10.3389/fcell.2023.1274807

**Published:** 2023-12-13

**Authors:** Valentina Woronzow, Jonas Möhner, Daniel Remane, Hans Zischler

**Affiliations:** ^1^ Division of Anthropology, Institute of Organismic and Molecular Evolution, Faculty of Biology, Johannes Gutenberg University Mainz, Mainz, Germany; ^2^ HOX Life Science GmbH, Frankfurt, Hessen, Germany

**Keywords:** cellular senescence, SVA, retrotransposition, somatic mosaicism, RDA, numts, mtDNA

## Abstract

Cellular senescence is characterized by replication arrest in response to stress stimuli. Senescent cells accumulate in aging tissues and can trigger organ-specific and possibly systemic dysfunction. Although senescent cell populations are heterogeneous, a key feature is that they exhibit epigenetic changes. Epigenetic changes such as loss of repressive constitutive heterochromatin could lead to subsequent LINE-1 derepression, a phenomenon often described in the context of senescence or somatic evolution. LINE-1 elements decode the retroposition machinery and reverse transcription generates cDNA from autonomous and non-autonomous TEs that can potentially reintegrate into genomes and cause structural variants. Another feature of cellular senescence is mitochondrial dysfunction caused by mitochondrial damage. In combination with impaired mitophagy, which is characteristic of senescent cells, this could lead to cytosolic mtDNA accumulation and, as a genomic consequence, integrations of mtDNA into nuclear DNA (nDNA), resulting in mitochondrial pseudogenes called *numts*. Thus, both phenomena could cause structural variants in aging genomes that go beyond epigenetic changes. We therefore compared proliferating and senescent IMR-90 cells in terms of somatic *de novo numts* and integrations of a non-autonomous composite retrotransposons - the so-called SVA elements—that hijack the retropositional machinery of LINE-1. We applied a subtractive and kinetic enrichment technique using proliferating cell DNA as a driver and senescent genomes as a tester for the detection of nuclear flanks of *de novo* SVA integrations. Coupled with deep sequencing we obtained a genomic readout for SVA retrotransposition possibly linked to cellular senescence in the IMR-90 model. Furthermore, we compared the genomes of proliferative and senescent IMR-90 cells by deep sequencing or after enrichment of nuclear DNA using AluScan technology. A total of 1,695 *de novo* SVA integrations were detected in senescent IMR-90 cells, of which 333 were unique. Moreover, we identified a total of 81 *de novo numts* with perfect identity to both mtDNA and nuclear hg38 flanks. In summary, we present evidence for possible age-dependent structural genomic changes by paralogization that go beyond epigenetic modifications. We hypothesize, that the structural variants we observe potentially impact processes associated with replicative aging of IMR-90 cells.

## 1 Introduction

Cellular senescence has received increasing attention as a fundamental factor in chronic disease and functional decline in older age. Senescent cells enter a permanent growth arrest after surpassing a limited number of cell divisions while remaining metabolically active (Hayflick and Moorhead, 1961). They are characterized by a whole range of different features, including disturbances in mitochondrial dynamics—changes in mitochondrial morphology due to fission and fusion processes, and both epigenetic and genetic alterations, e.g., phenomenons linked to telomere attrition. While some characteristics are considered beneficial—such as remodeling features and tumor suppression—senescent cells accumulate in aging tissue and take part in inflammation responses and tumorigenesis, actively losing their regenerative capacities (Li et al., 2020; Farfariello et al., 2022). Epigenetic changes such as the loss of repressive constitutive heterochromatin and subsequent LINE-1 derepression (Simon et al., 2019), as well as faulty mitochondrial quality control mechanisms have the potential to give rise to somatic structural genomic variants in senescent cells. The former phenomenon triggering mobilization and possibly insertional mutagenesis of both autonomous LINEs and non-autonomous SINEs. In addition, escape of mtDNA from mitochondria is usually associated with mitochondrial damage and mitophagy, which is impaired in senescent cells *in vitro* and *in vivo* (Dalle Pezze et al., 2014; García-Prat et al., 2016), possibly leading to cytosolic mtDNA accumulation and as a genomic consequence to integrations of mtDNA into nuclear DNA (nDNA), resulting in mitochondrial pseudogenes called *numts*. Thus, both sorts of DNA mobilization connect to age-associated hallmarks as summarized by López-Otín et al. (2023) and potentially cause structural variants. The upregulation of TE-expression—mainly autonomous LINE-1—in senescent cells has been described (De Cecco et al., 2019) and repeatedly confirmed. Moreover, several reporter-based approaches to detect somatic integrations of non-autonomous retrotransposons (e.g., SVA, Hancks et al. (2011)), together with qPCR approaches to distinguish copy number variations in young and aged cells of Alu-SINEs suggest that somatic TE integrations tissue-specifically increase as cells age (Morgan et al., 2017). A genome-wide qualitative and quantitative analysis of the genomic consequences of TE-derepression and mobilization, i.e., the landscape of somatic *de novo* integrations during senescence of a cellular model system to study *in vitro* cell aging such as human IMR-90 cells (Sherwood et al., 1988) is still pending. We therefore compared proliferating and senescent IMR-90 cells, the latter phenotypically defined by the senescence associated secretory phenotype (SASP), with respect to somatic *de novo numts* and integrations of a non-autonomous retrotransposon—the so called SVA-elements - that hijack the retropositional machinery of LINE-1. The SINE-VNTR-Alu retroposons are the “youngest” class of hominoid-specific TEs, preferentially found in gene- and GC-rich regions and possibly co-regulating nearby genes. Since they exhibit high mobility in the human germline, we investigated whether this is also reflected in somatic cells and during the processes of senescence due to replicative exhaustion. Due to the rarity of these *de novo* structural variations caused by retropositions, we used a representational difference analysis approach (RDA, Lisitsyn et al. (1993)). This subtractive and kinetic enrichment technique, that is able to detect small differences between complex genomes, was coupled with deep sequencing. More precisely, we PCR-amplify the upstream flank of the 5′SVA region using SVA outward primers in combination with flank-adaptors and thus generate a representation of SVA flanks in proliferating and senescent IMR-90 cells. The sample of proliferating cells was used in a 100-fold excess as driver, denatured and hybridized to the tester senescent DNA that was linked to an RDA-adaptor and subsequently used as PCR template. The resulting products were deep sequenced and bioinformatically checked with a customized pipeline, as outlined in our previous work (Möhner et al., 2023). Our ultimate goal was to get both qualitative—such as target site characteristics—and semi-quantitative information on the senescence associated SVA retropositions, the latter mainly because these integrations are regarded as rare genomic changes (Rokas and Holland, 2000) without exhibiting reversals and parallelisms in different cell lineages. To further extend our investigation of mobile DNA such as SVAs, we implemented a comparative method to detect possible interorganellar transfer of DNA between mitochondria—characterized by senescence-related mitochondrial dysfunction associated with impaired mitophagy (Korolchuk et al., 2017)—and the nucleus. Total DNA of proliferating and senescent IMR-90 cells were whole genome sequenced and in addition, we followed a strategy of specifically enriching nuclear sequences applying an AluScan PCR with primers anchored in Alu-SINEs as described by Mei et al. (2011). The resulting Inter-Alu representations of both proliferating and senescent nuclear genomes were then NGS-sequenced. Results of both experimental approaches were bioinformatically scanned for sequences that perfectly matched both mtDNA—with the fast evolving D-loop sequences specifically acquired from our IMR-90 sequencing data—and flanking nuclear DNA in a continuous 150 bp read of the WGS and the AluScan NGS data. The obtained sequences, containing both mtDNA and nDNA, where then filtered to exclude hg38-annotated *numts* (UCSC) and checked for possible target site patterns.

## 2 Materials and methods

### 2.1 Sample preparation: cell culture and SA-β-gal activity

Human fetal lung fibroblasts (IMR-90) were cultivated in Dulbecco’s modified Eagle’s medium (DMEM, Gibco™ Thermo Fisher Scientific, 11965092), supplemented with 10% fetal bovine serum (FBS, Gibco™ Thermo Fisher Scientific, A5256701). Proliferating IMR-90 cells were used as controls (PDL ≤30) and cultured until replicative exhaustion is reached to obtain senescent IMR-90 cells. Cell growth was assessed by calculating population doubling level of each cell passage (Supplementary Figure S1A). Senescence-associated β-galactosidase activity was estimated using an adapted staining protocol (Supplementary Figure S1B, C). Senescent cells were collected when cell proliferation ceased for at least a week (PDL ≤0.1) and the subsequent senescence-associated β-galactosidase assay was positive. In general, the collection was done no more than 3 weeks after the identification of the growth stagnation in the culture. The PDLs for each senescent sample in this analysis can be found in Supplementary Table S2. Proliferating and senescent cells were harvested using 1X PBS washing solution and 0.05% trypsin-EDTA (Gibco™ Thermo Fisher Scientific, 25300104).

### 2.2 DNA isolation

Cells were harvested (5 × 10^6^ per sample), resuspended in 1X PBS and treated with RNAse A (20 mg/mL) for 20 min at 37°C. Enzyme inactivation was performed with Proteinase K (40 mg/mL). DNA isolation steps were completed according to the QIAamp^®^ DNA Mini Kit (Qiagen, 51304).

### 2.3 RNA isolation

Harvested cells (1–5 × 10^6^ per sample) were resuspended in ZymoResearch^©^ RNA Lysis Buffer and treated according to manufacturer’s protocol (Quick-RNA Miniprep Kit, ZymoResearch^©^, R1055). By adding 2:1 EtOH (>99%) to the lysate sample at the column binding step, smaller RNA fragments were enriched.

### 2.4 RNA-sequencing

2 µg total RNA of proliferating and senescent IMR-90 cells were 150 paired-end mRNA sequenced, resulting in raw data as FASTQ files provided by Novogene Co., Ltd. using the Illumina NovaSeq 6,000 platform.

#### 2.4.1 Differential expression analysis

The provided transcriptomic data of proliferating and senescent IMR-90 cells was further analyzed by quantifying transcript expression into read count files using Salmon (Patro et al., 2017), transforming the generated transcript-level abundancies to gene-level estimates utilizing tximport (Soneson et al., 2015) and finally performing differential expression analysis using DESeq2 (Love et al., 2014). Fold changes and *p*-values were checked for genes of interest, including mitochondrial clearance, integrity and mitophagy genes and genes in close relation to open chromatin, cell proliferation and senescence as listed in the SASP (R-HSA-2559582; Gillespie et al., 2022; Supplementary Table S1.1) and the SenMayo gene set (Saul et al., 2022; Supplementary Table S1.2).

#### 2.4.2 Local BLAST+ of transcriptomic data

FASTQ files of proliferating and senescent IMR-90 cell transcriptomes were mapped to the human genome (hg38) using Bowtie2 (Langmead and Salzberg, 2012). SAM files generated in this way were transformed to sorted BAM files and finally to FASTA files containing only mapped reads using samtools (Danecek et al., 2021). Next, we ran local BLAST analyses of the transcriptome data with SVA query sequences to evaluate retrotransposon RNA abundance. To this end, we obtained SVA subfamily consensus sequences (SVA_A to SVA_F) from the DFAM database (https://dfam.org, Storer et al., 2021). The SVA subfamily sequences were aligned and checked for subfamily-specific informative regions. To minimize redundant hits due to the composite and tandem repetitive character of the SVA sequence, a 200 nt-query from the 5’ region of the SVAs was chosen as query sequence. Blastn-settings included -perc_identity 95 to retrieve exclusively 95% identical hits. Hits were corrected for the overall alignment rate in the respective datasets as determined by Bowtie2.

### 2.5 Representational difference analysis (RDA) of SVAs

Representational difference analysis including laboratory steps such as PCR, hybridization and RDA amplifications and bioinformatical steps were performed as previously described (Möhner et al., 2023). 100 ng DNA obtained from proliferating (used as driver) and senescent (used as tester) IMR-90 cells were applied, respectively. 150 paired-end NGS data (FASTQ files) of the final PCR products including possible *de novo* insertions of SVAs in senescent IMR-90 cells (*n* = 3) were generated on the Illumina NovaSeq 6,000 platform by Novogene Co., Ltd.

#### 2.5.1 Genomic target site and motif analysis of SVA integrations

Genomic target site coordinates of *de novo* SVA insertions were extracted as BED files applying a customized bioinformatical pipeline as previously described (Möhner et al., 2023). We used the HOMER software to detect specific target site features (AnnotatePeaks.pl). Motifs were analyzed using MEME Suite (http://meme-suite.org/) with default settings to identify possible L1 cleavage sites in the *de novo* insertion flanks (Bailey and Elkan, 1994).

### 2.6 Human whole genome sequencing (hWGS)

1.5 µg total DNA was isolated from proliferating and senescent IMR-90 cells as previously described (2.2) and library preparation and sequencing was performed with a 3X coverage by Novogene Co., Ltd. on the Illumina NovaSeq 6,000 platform and PE150 strategy obtaining raw reads as FASTQ files.

### 2.7 AluScan-PCR and next-generation sequencing

To reduce the background of multicopy mtDNA, we additionally applied an “Alu-anchored scan” or “AluScan” to enrich for nuclear DNA sequences between Alu-sequences. This PCR method was described by Mei et al. (2011) and uses primers that bind to specific Alu-elements, the most abundant, Short Interspersed Nuclear Elements (SINEs) in the human nuclear genome exceeding a million of copies. We used the AluY278T18-, AluY66H21- and R12A267-primers according to the protocol outlined in Mei et al. (2011) and performed a PCR starting from 100 ng input DNA of both proliferating and senescent cells with 35 cycles using the Qiagen PCR Taq Core Kit (201,223, Qiagen). The resulting heterogeneous PCR products were then purified using the QIAquick PCR Purification Kit (28,104, Qiagen) and deep sequenced (150 PE) by Novogene Co., Ltd. on the Illumina NovaSeq 6,000 resulting in FASTQ files that were subsequently bioinformatically processed as outlined below.

### 2.8 Bioinformatical scanning of *de novo numt*s

To investigate possible *de novo* insertions of mtDNA into the nuclear DNA, a customized BASH script was established to detect *numt*-flanking regions in senescent and proliferating IMR-90 cells. For all extracted FASTA files from the hWGS and AluScan-Seq data a BLAT search with the complete IMR-90 mtDNA and the fast-evolving D-Loop, which was assembled from our WGS data, was performed (Kent, 2002). By utilizing the UCSC-PslScore script, output files with 100% identical mtDNA hits of all data sets were filtered with respect to the alignment coverage. Each alignment exceeding 130 nt in length was discarded to finally get informative genomic flanking sites of a minimum size of 20 nt. Mitochondrial alignment fractions were then subtracted from the whole sequence reads and the remaining sequence read of ≥20 nt in length was mapped to the human genome hg38 using BLAT to search for possible integration sites. By using the UCSC-cDNAFilter we then extracted alignments with a minimum query length of 95%, uniquely mapped reads, gapless alignments and further deleted any hits that contained remaining mitochondrial segments in the flanking sequences. The BED files of proliferating IMR-90 data sets generated in this way—containing filtered genomic positions of possible *de novo numt* target sites—are then subtracted from the senescent IMR-90 data sets utilizing BEDtools intersect (Quinlan and Hall, 2010). By that, we managed to obtain target sites specific to senescent IMR-90 cells. Those BED files were then further processed by subtracting known genomical coordinates of *numts* (hg19 liftover to hg38) and further checking every read associated with a possible *de novo numt* by performing a NCBI BLAST search throughout all known genomical databases for taxid 9,606 (human), thus excluding already annotated *numts.*

#### 2.8.1 Genomic target site analysis of *de novo numts*

We further analyzed *de novo numts* of senescent IMR-90 cells by checking which mitochondrial genes were recruited for the integration and further, which genomic target sites were affected by the integration process. We first applied a BLAST alignment for all mapped mtDNA segments in our final *de novo numts* FASTA files and annotated their mitogenomic features. For the flanking regions–excluding mtDNA related fractions—we utilized HOMER to annotate the coordinates of the integrations on hg38 (AnnotatePeak.pl).

## 3 Results

### 3.1 RNA abundance of SVAs and gene expression changes associated with chromatin remodeling in senescent IMR-90 cells

**FIGURE 1:**
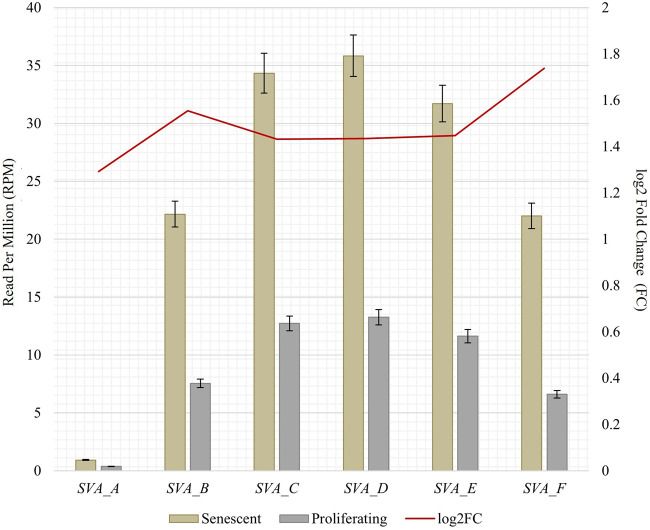
RNA abundance of SVA_A-SVA_F in senescent and proliferating IMR-90 cells, determined by NCBI BLAST+ (*blastn*) alignment of 5′ SVA sequences (200 nt) to *bowtie2* mapped transcriptomic data. Normalized aligned reads (*n* = 2) depicted as Reads Per Million (RPM; left y-axis) with standard error shown as bars and log_2_ Fold Change (FC; right y-axis).

**FIGURE 2:**
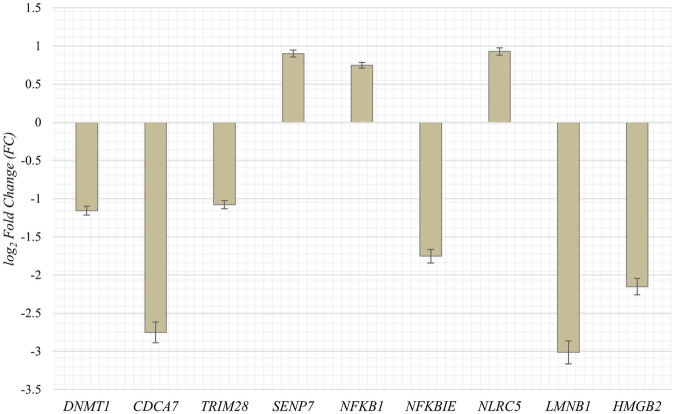
Expression of genes associated with cell proliferation and chromatin remodeling in senescent IMR-90 cells (*n* = 2). Log_2_ Fold Changes (y-axis) depicted for nine GOI with standard error displayed as bars (x-axis).

To obtain RNA sequencing data for both proliferative and senescent IMR-90 cells, a 150 PE strategy was applied. First, we determined the overall alignment rate of our transcriptome data using bowtie2. Second, we investigated the overall expression of SVAs and thus the availability of SVA transcripts for the onset of SVA mobilization in IMR-90 cells. To this end, we investigated the expression of SVA_A—SVA_F in our transcriptome data from proliferating and senescent IMR-90 cells using the consensus sequences of the SVA subfamilies as queries. More precisely, the 5′sequences of the six SVA subtypes A-F were truncated to a length of 200 nt, and we used NCBI BLAST+ (blastn) to align them to the hg38-mapped IMR-90 transcriptome. While the first 200 nt of SVA_A showed the lowest read count alignment (senescent: 0.92 RPM; proliferating: 0.37 RPM), we detected an overall increase in SVA expression with log_2_ fold changes ranging from 1.29 in SVA_A to 1.74 in SVA_F for the senescent datasets (Figure 1). Interestingly, the most normalized read counts were found for SVA_D (36 RPM with an FC of 1.43), SVA_C (34 RPM with an FC of 1.43), and SVA_E (32 RPM with an FC: 1.45). Beside the mobilization of TEs, a successful reintegration is controlled and regulated by a variety of different factors, among them the methylation of CpGs. Both the dynamics of chromatin and changes of transcriptional networks are central with respect to aging associated changes (De Cecco et al., 2013). To get a first idea of the age-associated dynamics related to chromatin remodeling, we examined the presumably epigenetically controlled transcript abundance of genes involved in chromatin remodeling and cell proliferation in the transcriptome data of proliferative and senescent IMR-90 cells. By performing differential expression analysis, we observed a decrease in *DNMT1* transcripts in the senescent IMR-90 data (FC: −1.16), followed by *CDCA7* (FC: −2.75), *LMNB1* (FC: −3), and *HMGB2* (FC: −2.15; Figure 2). We hypothesize that downregulation of those genes that are associated with increased chromatin remodeling dynamics might increase chromatin accessibility in replicative senescent IMR-90 cells.

### 3.2 Active *de novo* SVA retrotransposition in cellular senescent IMR-90 cells

**FIGURE 3:**
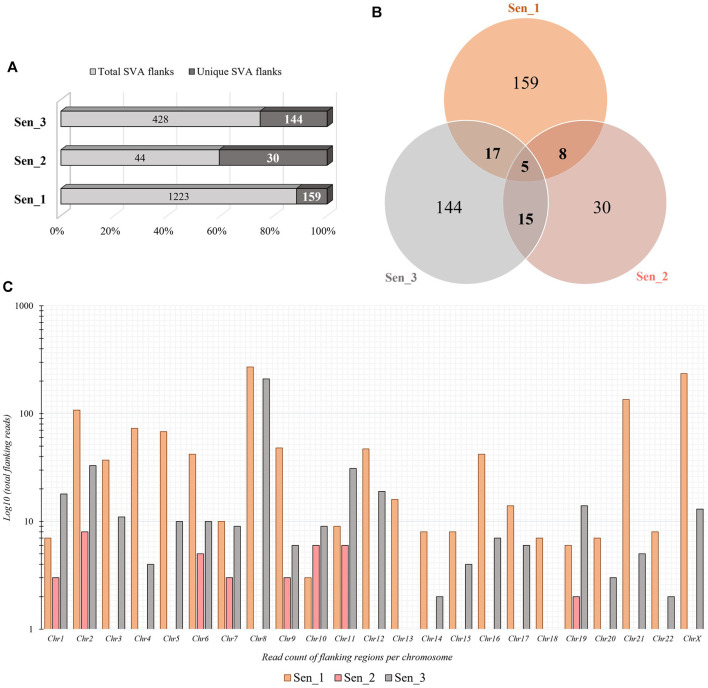
Quantity and chromosomal distribution of somatic *de novo* integrations of non-autonomous SVAs in senescent IMR-90 cells (*n* = 3). **(A)** Total and unique SVA integration flanks in the three samples (Sen_1—Sen_3) displayed in % shown as bars. **(B)** Venn diagram of the unique, shared SVA integrations between all three RDA samples (Sen_1—Sen_3). **(C)** Distribution of chromosomal SVA flanking regions (Sen_1—Sen_3) displayed as log_10_ of total read counts (y-axis) per chromosome (x-axis).

Somatic *de novo* integrations of non-autonomous SVAs in senescent IMR-90 cells were investigated using the RDA enrichment technique (Lisitsyn et al., 1993), with tailored modifications and additional deep sequencing techniques (Möhner et al., 2023). Three batches of replicative senescent IMR-90 cells (Sen_1–3) were treated accordingly as tester samples to identify novel integrations occurring during the process of senescence. Proliferating IMR-90 cell DNA served as driver samples that represent 5′-flanks of both evolutionary fixated SVA integrations and polymorphic ones. Only the tester samples are covalently ligated to specific RDA primers, which ensured an exponential enrichment of SVA flanks that are absent in the driver sample. The resulting PCR-enriched and deep sequenced SVA flanks were analyzed using a customized bioinformatical pipeline. To scan for novel integration properties, previously annotated SVAs (hg38) were filtered out from the data sets and the remaining flanking sequences were mapped to the human genome (hg38) using BLAT v.36 (Kent, 2002), to determine specific coordinates for novel SVA insertion events and their genomic target sites. Overall, we detected a total of 1223 SVA insertions in the first sample (Sen_1), of which 159 hg38 flanking coordinates were unique. The second sample (Sen_2) contained 44 total and 30 unique SVA flanks and 428 total and 144 unique were discovered in the third sample (Sen_3), respectively. We then checked for shared positions of *de novo* integrations and found five SVA target site coordinates which showed an overlap between all three samples (Figures 3A, B). Global chromosomal distribution was analyzed using the positional BED files containing all SVA flanks in the three data sets. Total read counts of *de novo* SVA flanks were logarithmically corrected and possible differences and similarities have been featured between the three samples. As for Sen_1, most flanking regions were detected on chromosome 8 (271 flanks), followed by chromosome X (235 flanks) and chromosome 21 (135 flanks). Sen_3 shares the most *de novo* integration flank-counts with Sen_1 on chromosome 8, with a total abundancy of 210 flanking sequences. In addition, for the third sample, 33 flanks are found on chromosome 2 and 31 flanks on chromosome 11. Sen_1 and Sen_3 exhibit multiple target site locations scattered across the entire genome, resulting in a highly diverse landscape of SVA integrations (Figure 3C). These findings suggest ample evidence for active SVA retrotransposition in the context of cellular senescence and highlight that the herein presented senescence-associated abundance of chromosomal targets deviates from known SVA integration patterns. Overall genomic density of germline SVAs in humans is more prominent on chromosomes 1, 17, 19, and 22 (Wang et al., 2005), whereas our results on somatic and age-associated SVA retropositions suggest integration preferences on chromosomes 2, 8, 12, 21, and X in senescent IMR-90 cells.

#### 3.2.1 Genomic target sites of *de novo* SVA integrations

**FIGURE 4:**
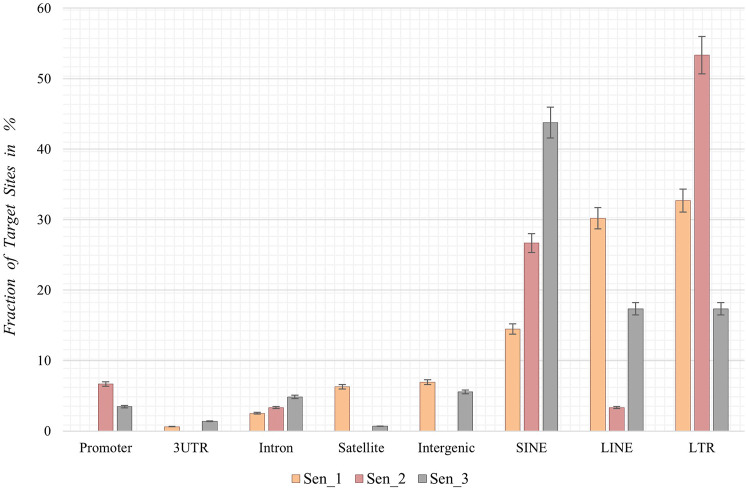
Annotation of genomic features of *de novo* SVA integrations in senescent IMR-90 cells (Sen_1—Sen_3; *n* = 3). Annotated features with respect to the sum of all HOMER-annotated features (x-axis) for each sample displayed as % fraction with standard error (y-axis) of all target sites. Features on x-axis are (from left to right): Promoter-TSS from −1 kb to +100 bp (Promoter), 3′-untranslated region (3UTR), intronic region (Intron), satellite region (Satellite), intergenic region (Intergenic), SINE transposons (SINE), LINE transposons (LINE) and long terminal repeats (LTR).

Following the chromosomal distribution analysis, target site coordinates of the three samples were annotated using HOMER software, thereby elucidating preferred locations with respect to genomic features and preferred locations of senescence-associated integrations. Interestingly, retrotransposons such as LINEs, SINEs, and LTRs appear to be favored targets for *de novo* SVA insertions in all three senescent data sets. Sen_1 exhibits most annotated integration targets in LTRs (32.7%, Figure 4), followed by LINE-1 with 30.2% and SINEs in form of Alu elements (14.5%). With 53.3%, Sen_2 displays most integration targets in LTR regions, followed by Alu SINEs (26.7%) and promoter regions (6.7%). The third replicate reveals most target sites in Alu-SINEs (43.8%), LINE-1 (17.4%) and LTRs (17.4%). In summary, *de novo* SVA insertions in senescent IMR-90 cells take place in regions already affected by previous retrotranspositions, next to a less pronounced fraction located in intronic [2.5% (Sen_1), 3.3% (Sen_2), 4.9% (Sen_3)] and intergenic [6.9% (Sen_1) and 5.6% (Sen_3)] regions (Figure 4).

### 3.3 Cytosolic mtDNA accumulation in cellular senescent IMR-90 cells

**FIGURE 5:**
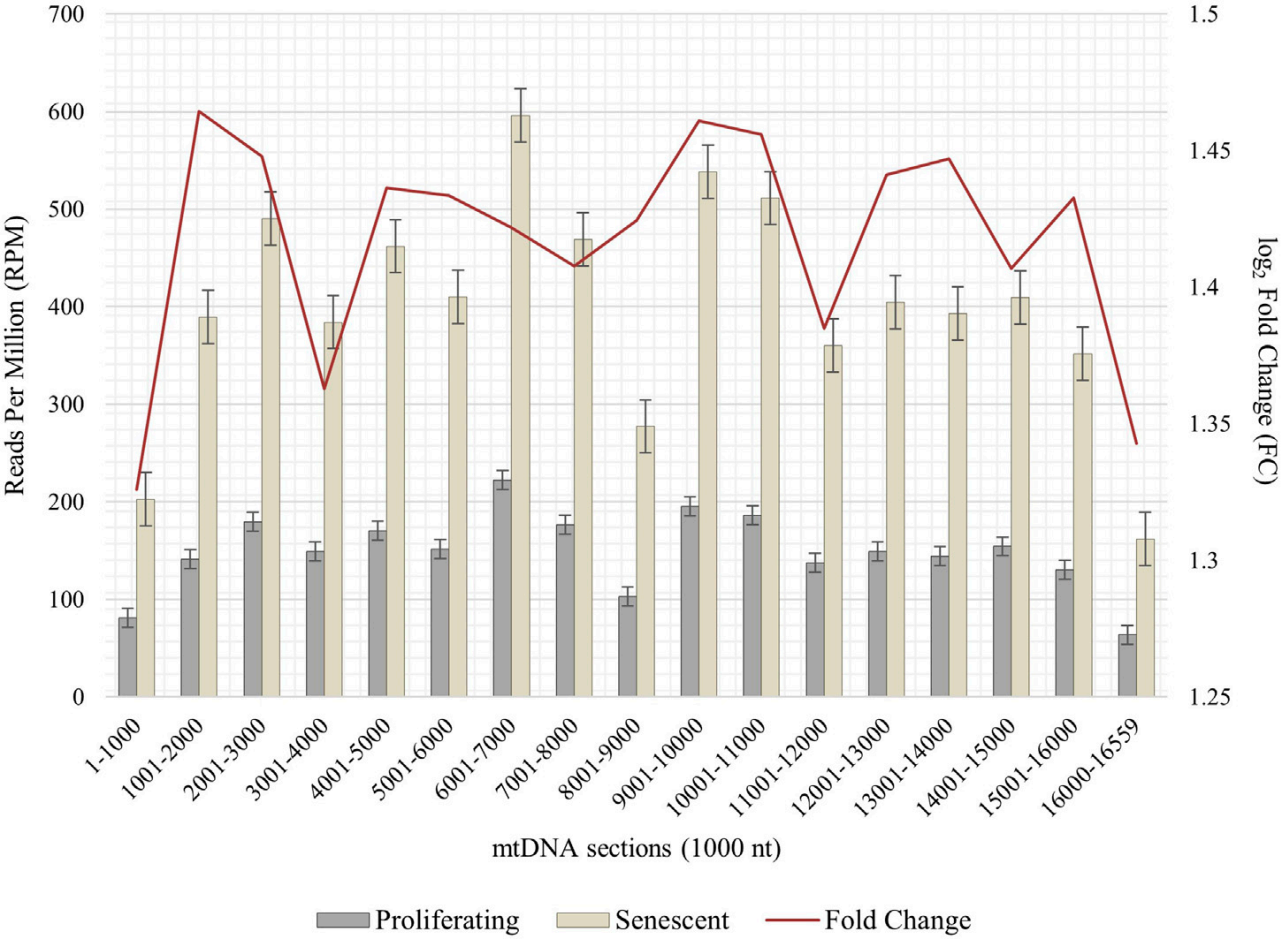
Abundance of mtDNA copies in proliferating and senescent IMR-90 cells. mtDNA sequence (D38112 with additional IMR-90 cell-specific D-Loop) is sectioned in 1,000 nt fractions from 1–16,559 bp (x-axis), aligned read counts (RPM, left y-axis) are displayed as bars with standard error for proliferating and senescent data, fold change (FC) is depicted as a secondary graph overlay (y-axis).

Due to the importance of senescence-related mitochondrial dysfunction associated with impaired mitophagy and mitochondrial clearance processes, we asked whether an interorganellar transfer of mobile DNA—more precisely the paralogization of mtDNA to *numts*—is characteristic for cellular senescent IMR-90 cells. Total DNA of proliferating and senescent cells were whole genome sequenced and bioinformatically processed (*n* = 2). Prior to detecting a possible interorganellar transfer of DNA between mitochondria and the nucleus, we checked for mtDNA quantity and overall distribution in our WGS data. Whole mitochondrial DNA (D38112) was thus split into 1,000 nt subsequences and the fast-evolving D-Loop sequence was assembled from our WGS data. These sequences were used as queries in a local NCBI BLAST+ (*blastn, 100% perc_identity*) to analyze proliferating and senescent WGS databases. Overall, the mtDNA read counts (reads per million, RPM) show a substantial increase ranging from 1.3 to 1.46 FC (fold change) in the senescent samples throughout all 1,000 nt mtDNA subranges (Figure 5). Lowest relative read counts were observed for the mitochondrial genome position 8,001–9,000 nt (senescent: 277 RPM; proliferating: 103 RPM), where the transcript for ATPase subunit 6 (7,941–8,621 nt) and cytochrome c oxidase subunit 3 are located (8,621–9,404 nt). We detected the highest read counts for the query positions 6,000–7,001 nt (senescent: 596 RPM; proliferating: 222 RPM) including the sequences for cytochrome c oxidase subunit 1 (5,327–6,868 nt), tRNA-Ser (6,868–6,939 nt) and tRNA-Asp (6,941–4,008 nt).

### 3.4 Cellular senescence drives *de novo* nuclear integration events of mtDNA

**FIGURE 6:**
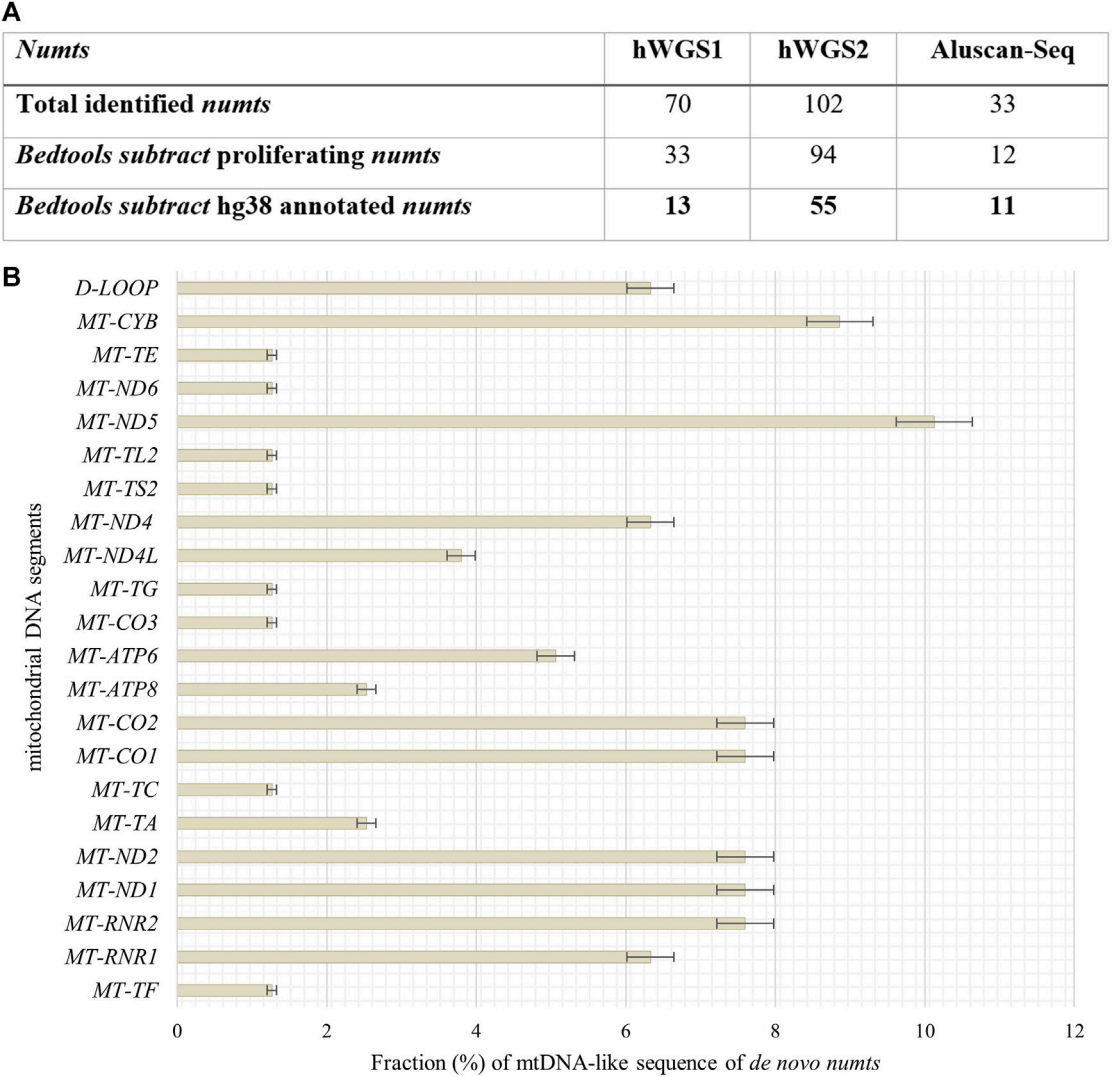
Quantity of identified *numts* and disitribution of their mtDNA like segments in senescent IMR-90 cells. **(A)** Table of all *numts* (total read counts) identified in the three data sets: hWGS1, hWGS2 and Aluscan-Seq. **(B)** Abundance and distribution of mtDNA-like segments of all *de novo* *numts* (=79) as % with standard error (y-axis), displayed as bars.

To define *de novo* transferred mtDNA in the nucleus of senescent IMR-90 we applied a customized bioinformatical pipeline, defining full length-reads with both 100% identity to IMR-90-mtDNA and hg38 genomic DNA. To extract genomic coordinates of *de novo numts* target sites we analyzed two standard WGS datasets for each the proliferating and senescent IMR-90 cells. In addition, we enriched the nuclear genome information against a background of the multycopy-mtDNA by amplifying the regions between Alu-SINES, the most abundant SINE with respect to copy number in the human genome. To this end, the AluScan method (Mei et al., 2011) was applied as suggested by the authors and the amplificates were deep sequenced. All NGS results were scanned for mtDNA insertions by reads partially consisting of perfect identical mtDNA and nuclear flank sequence. By eliminating known hg38-annotated *numts* and intersecting the *numt* coordinates of the senescent sample with genomic *numt* coordinates of the proliferating sample, we excluded germline-specific *numts* within IMR-90 cells and obtained unique target sites that can be further investigated in the context of cellular senescence. In total, we managed to identify 79 *de novo numts* in senescent IMR-90 of which 13 were found in the first WGS data set, 55 in the second WGS data set and 11 in the AluScan Seq data (Figure 6A).

#### 3.4.1 mtDNA mobility and *numts*

We identified 79 *de novo numts* with perfect identity to mtDNA and nuclear hg38 flanks in senescent IMR-90 cells and thus wanted to further evaluate the abundance and distribution of specific mtDNA regions, i.e., a potential preferential mobility of certain mtDNA regions. Therefore, mtDNA-like sequence fractions of our data were annotated by alignment to the mitochondrial genome (D38112) complemented by the IMR-90 specific D-Loop sequence. 6% of these mtDNA-like sequence fractions within our *de novo numts* represent paralogs of the D-Loop (Figure 6B). Most abundant mtDNA fragments of *de novo numts* belong to *MT-ND5* with 10%, followed by *MT-CYB* (9%) and equally *MT-RNR2, MT-ND1, MT-ND2, and MT-CO1. MT-CO2* (8%). Further, MT-RNR2, MT-ND1, and MT-ND2 share the same feature count in our senescent data (7.6%).

#### 3.4.2 Mitochondrial quality control and somatic interorganellar transfer

**FIGURE 7:**
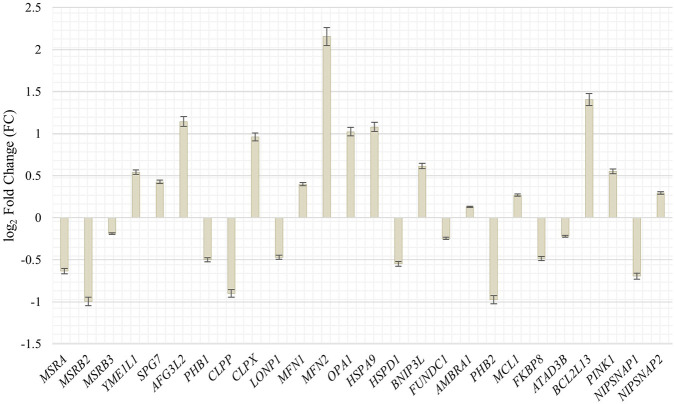
Expression of genes associated with mitochondrial integrity, clearance and mitophagy in senescent IMR-90 cells (*n* = 2). Log_2_ Fold Changes (y-axis) depicted for 26 GOI with standard error displayed as bars (x-axis).

To get further insight in the integrity and quality control of mitochondria during the senescence process and the possible association with an increased mobility during senescence, we investigated genes and their abundancy in proliferating and senescent IMR-90 cells associated with mitochondrial integration, clearance and mitophagy. Specific genes, that might be associated with mtDNA accumulation and depletion in the context of cellular senescence, were chosen (Szklarczyk et al., 2014; Srinivasainagendra et al., 2017; Livingston et al., 2019). Differential expression analysis of the transcriptomic data of proliferating and senescent IMR-90 cells (*n* = 2) was performed and fold changes checked for the GOI. In total, 27 genes associated with mitochondrial pathways were checked for differences in expression. Interestingly, while mRNA could be observed for all genes in the proliferating and senescent data sets, we did not detect any significant expression differences except for three genes (Figure 7). As for the mitochondrial integrity genes, *MSRB2* expression is decreased in senescent cells (FC: −1; *p* ≤ 0.05) whereas *MFN2* expression is highly increased (FC: 2.2; *p* ≤ 0.05). Additionally, one gene of the mitochondrial clearance family is enriched in senescent cells: *BCL2L13* (FC: 1.4; *p* ≤ 0.05). Beside the above-mentioned accumulation of mtDNA during senescence, we thus observed changes in the expression for several genes actively involved in mitochondrial homeostasis in senescent IMR-90 cells.

#### 3.4.3 Target sites of *de novo numts* in senescent IMR-90 cells

**FIGURE 8:**
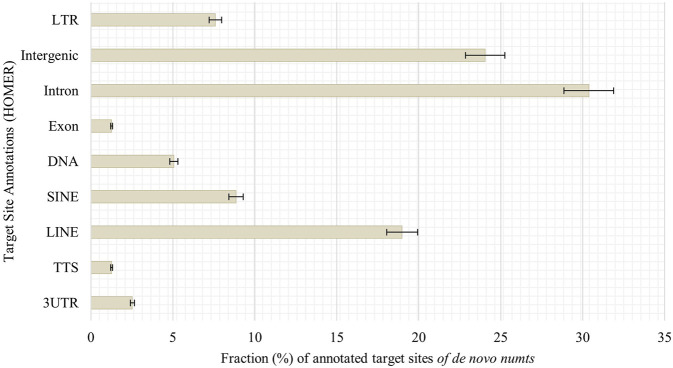
Annotation of genomic features of *de novo numts* in senescent IMR-90 cells. Annotated features with respect to the sum of all HOMER-annotated features (y-axis) for each sample displayed as % fraction (x-axis) of all target sites. Features on y-axis are (from top to bottom): long terminal repeats (LTR), intergenic region (Intergenic), intronic region (Intron), exonic region (Exon), DNA, satellite region (Satellite), SINE transposons (SINE), LINE transposons (LINE), transcription termination site from −100 bp to +1 kbp (TTS) and 3′-untranslated region (3UTR).

Looking into possible target sites of senescence-associated *de novo numts*, we utilized HOMER AnnotatePeaks.pl to obtain peak annotations of the genomic coordinates. Most target sites can be found in introns (30%), followed by intergenic regions (24%), LINEs (19%) and SINEs (9%, Figure 8).

## 4 Discussion

Consistent with the data obtained for human LF1 diploid fibroblast cells (De Cecco et al., 2019), we observe a transcriptional upregulation of SVA elements in IMR-90 cells, too. First, our RNAseq-data comparing proliferative and senescent IMR-90 mirror a background expression in proliferative cells with an increase in SVA message in senescent cells. To take the SVA diversity into account and avoid misalignments due to the internally repetitive structure of SVAs, we differentially screened for RNA of the different SVA families. Querying with the informative 5′portion of SVA, we searched the RNA-databases, correcting for the overall alignment rate. Though the SVA elements in general were transcriptionally upregulated, we observed that this upregulation preferably affects SVA_F most strongly. Previous research elucidated that SVA_B and SVA_D are the most abundant within the human genome, a dependency of copy number and level of transcription is only mirrored for SVA_D-transcripts in our data (Wang et al., 2005). The human-specific SVA_A and SVA_F are expressed to a comparatively lower extent, however fold changes as obtained comparing proliferative and senescent cells suggest an accumulation of all six subfamilies in a senescence-associated manner.

Whether the abundance of the 5′SVA as described herein also reflects the diversity in the transcriptional units of SVAs mainly due to differing 5′TSS is not clear, since the preferred mode of SVA transcription is varying in different cell types (Hancks and Kazazian, 2010). A decrease in the methyltransferase *DNMT1* transcription (−1.16 FC) that might be linked to the process of senescence as described by Young and Smith (2001) could be observed in our data as well, pinpointing towards DNA methylation being effective to control SVA transcription. A possible pre-transcriptional control mechanism of SVA transcription might be CpG methylation, which could be however both systemic and specific at the same time and possibly dependent on the age of the TEs (Ewing et al., 2020). The nanopore sequencing approach by Ewing et al. (2020) has recently uncovered the complex nature of SVA methylation possibly influencing the spatial-temporal expression patterns of SVA and probably getting even more complex when considering age-associated changes. Therefore, we regard our obtained expression data as a rough proxy of potentially mobilizable SVA retroposons that increase in abundance with age. Our IMR-90 data thus recapitulate the observation of reactivated TEs which is observed across species (Bravo et al., 2020; Giordani et al., 2021). However, more research on differential DNA modifications concerning CpG methylation, accessible chromatin and histone modifications need to be conducted to get a more detailed understanding of the mechanisms of reactivated transposable elements and their targets. In the context of expression data, we additionally examined genes that play important roles in cell proliferation and global chromatin changes such as *LMNB1*—which is part of the nuclear lamina–and observed an overall decrease in transcript abundance in our senescent transcriptomic IMR-90 data (−3 FC). Downregulation of *LMNB1* is defined as a key trigger of chromatin remodeling as outlined by Shah et al. (2013). *HMGB2* encodes a chromatin-associated protein of the high mobility group family, and its’ expression is altered during replicative exhaustion as described by Aird and coworkers (2016). We observed a decrease for *HMGB2* (−2.15 FC) in the senescent IMR-90 transcriptome which is consistent with the observation of Aird et al. (2016). Finally, we detected a depletion of *CDCA7* (−2.75 FC) in the senescent transcriptomic data, which is closely linked to *DNMT1* expression and an overall hypomethylation at centromeric repeats as reviewed by Vukic and Daxinger (2019). Thus, our transcriptomic data of replicative exhausted IMR-90 cells provide preliminary indications for a possible dynamic remodeling of chromatin and thus a changed accessibility of reintegration target sites. However, we encourage further research on this issue to corroborate the possible link between accessible target sites and SVA-reintegration rates. For now, we focus more on the observed presence absence patterns that generate genomic variation between proliferative and senescent IMR-90 cells. De Cecco et al. (2013) hypothesized that the chromatin of repetitive elements becomes relatively more open during replicative exhaustion. We therefore asked whether this not only mobilizes non-autonomous SVA elements allowing a hijacking of the L1 machinery but also triggers *de novo* reintegration with respect to their genomic targets. To obtain maximum sensitivity in tracing *de novo* SVA integrations we applied an RDA based enrichment technique, in that we represented the 5′flanks of SVAs. The RDA is based on a hybridization of PCR fragments from two genomes under scrutiny—proliferative vs. senescent—in that driver 5′ SVA flanks from proliferating cells are given in excess and hybridized to senescent tester representations of 5′ SVA flanks, that were ligated to RDA-primers. Ideally, only fragments that originated in the senescent genomes could then be amplified exponentially thus finding the SVA-related differences between the genomes. The results from deep sequencing were scanned for fixated and germline transmitted SVA elements, thus hg38-annotated SVAs were excluded. Moreover, and since we perform intraindividual tester-driver comparisons, we also experimentally represent the fraction of presence/absence-polymorphic SVA-elements in both driver and tester increasing the specificity for *de novo* SVA integrations. In the three replicate experimental settings we could detect 1,223, 44, and 428 SVA *de novo* integrations from that 159, 30 and 144 were unique, respectively. The differing read counts and degree of mosaicism represent the individual unique events of SVA *de novo* integrations taking place at different PDL stages and on different lineages during somatic evolution towards senescence as each DNA sample input for the three batches was isolated from IMR-90 cells which underwent replicative exhaustion independently. Due to the low number of copies for each *de novo* insertion in all three replicates, with rates ranging from 90% to 100% for under 10 read counts per insertion, we assume a correlation between these insertions and the senescence status observed in the IMR-90 cells. SVA integrations represent rare genomic changes which has been observed in previous research (Raiz et al., 2012) and is further documented by the observation that of all detected *de novo* SVA insertions only five target site coordinates shared an overlap in our data. In conclusion and in an extension to the experiments with inducible and marked SVA retropositions (Hancks et al., 2011) we provide ample evidence for a marked genomic impact of mobile SVAs in IMR-90 cells that undergo cellular senescence. We present data as obtained by a highly sensitive and specific method to trace *de novo* formed SVA integration sites which showed that active *de novo* SVA mobilization, hijacking of the retrotranspositional machinery and reintegration is ongoing and frequently taking place in senescent IMR-90 cells. To come back to the status of open chromatin pertaining to the repetitive elements in senescent genomes, we next checked the localization of the *de novo* SVA integrations. To this end, BED-files with *de novo* integration coordinates were first annotated to hg38, revealing that LINEs, SINEs and LTRs are the preferred targets of *de novo* integration. To gain a better understanding of how the SVA insertions possibly integrate in our data, we analyzed L1 cleavage sites using the MEME discovery suite. This tool identifies the top five motif sites shared among the provided sequences. Our analysis shows that, among all functionally identified sites, 53% contribute to the motifs related to L1-cleavage sites in replicate Sen_2, 62% in Sen_3, and 64% in Sen_1 (Supplementary Figure S2). Additional motifs linked to the cleavage site were identified for Sen_1, with 28% and 11% of the sites contributing to these motifs. Flasch et al. (2019) report that L1 integration occurs frequently at degenerate consensus sequence sites, specifically 5′-TTTT/AA-3'. T-rich regions are commonly favored as insertion sites for L1, which we can partially transfer to our *de novo* SVA insertions. However, retrotransposition can arise in several ways outside the endonuclease activity of L1. Ono et al. (2015) describe *de novo* insertions found at DSB-introduced sites in mouse zygotes. This indicates that reverse transcription-product mediated double-strand break repair plays a role in integration events. Additionally, Morrish et al. (2002) reported the integration of L1 elements into DNA lesions at uncommon target sequences without endonuclease involvement. The L1 integrations exhibited 3′end truncations and lacked target site duplications. Further research is necessary to identify target sites of somatic *de novo* SVA integrations given the various mechanisms underlying retrotransposition. In an extension to the global patterns of *de novo* SVA integrations, we checked if they localize in close proximity to genes by checking the intronic or 5′and 3′regulatory regions as functionally annotated by HOMER. However, the low percentage of integration sites as depicted in Figure 4 and thus low quantity of targeted genes, highlight the importance of further experiments to solidify whether functional relevant integration patterns can be observed in a senescence-associated manner. Additionally, we tested whether our strategy is applicable for other transposable elements. Through a separate somatic mosaicism analysis, we discovered that our technique is not suitable for LINE-1 *de novo* insertions as we only obtained a very small number of LINE-1 integrations. We attribute this occurrence to a greater abundance of 5′-truncated LINE-1 elements. Our method relies on hybridization of PCR fragments obtained from 5′ flanks. The truncation phenomenon can be explained with the target primed reverse transcription, either by microhomologies (Zingler et al., 2005) or twin priming (Ostertag and Kazazian, 2001). A similar strategy for the 3′flank of LINE-1 is rather difficult to apply with respect to primer targets in specific regions, as variable sizes of poly(A) tails may cause problems in the accurate methodological conception. In our experience, the 5′flank amplification works well, the CCCTCT hexamer repeat might vary in its length–but on average, 30–50 repeats are observed. Thus, with 300–350 NGS fragmentation of the PCR products prior to sequencing, SVA flanks are still clearly definable with sizes of 50–100 bp. 5′-truncated SVAs have been observed as well, but overall, the hexamer repeat protects our outward primer targets due to its’ downstream position. On the other hand, outward primers specific for the LINE-1 elements, are located directly in the UTR, posing a difficulty. Regarding the identification of possible *de novo* Alu elements, the high copy number in the human genome and internal similarities can interfere with our specific hybridization method. The same applies to the subsequent bioinformatic assessment of *de novo* Alus, as the distinguishment from germline transmitted Alu integrations becomes increasingly complex due to the much higher copy number. Overall, with the lower abundancy of SVAs, their published mobility which we *bona fide* transpose to the somatic situation and our goal to identify essentially homopolasy-free genomic variation in the process of cellular senescence, we are confident that our approach gives solid information on structural variation caused by non-autonomous elements represented by the *de novo* SVA integrations.

It is generally accepted that cytoplasmic DNA is an efficient damage-associated molecular pattern (DAMP) that triggers the innate immune system responses associated with aging. Beside reverse transcribed autonomous and non-autonomous TEs, another source of cytoplasmic DNA arises upon release of mtDNA from the mitochondrial compartment. This release, among other things, serves as a rate-determining step for numtogenesis, as summarized by Singh et al. (2017). The mtDNA copy number, together with impaired mitophagy characteristic of old cells, contributes to the cytoplasmic mtDNA quantity possibly initiating the interorganellar transfer into the nucleus. Concordantly, studies in rats have shown that the amount of mtDNA in the nucleus increases with the age of individuals in liver and brain (Caro et al., 2010). Additionally, in a recent study conducted by Victorelli et al. (2023), the phenomenon of MOMP (mitochondrial outer membrane permeabilization)—a newly discovered key feature of cellular senescence associated with mitochondrial apoptotic stress without a resulting cellular death–was described to drive the release of mitochondrial DNA into the cytosol of aged mice. Therefore, we first checked in our hWGS data whether we can observe an increase in mtDNA copy number associated with IMR-90 senescence. To this end, we queried the WGS data of proliferating and senescent IMR-90 cells (*n* = 2) with mtDNA sequences partitioned into 1000 nt fragments and with the rapidly evolving D-loop sequences assembled from our IMR-90 WGS data. By comparing proliferative and senescent cells, we obtained evidence for an increase in mtDNA copy number in senescent cells. A decrease in mtDNA copy number, or mtDNA depletion, is seen in many cancers (Abd Radzak et al., 2022). Vice versa, it has been speculated that the accumulation of mtDNA in senescent cells may be a key factor in suppressing tumorigenesis or inflammatory responses (Miwa et al., 2022). This positive effect could be counteracted by increased leakage of mitochondrial genetic material first into the cytoplasm and being eventually transferred into the nucleus. Several scenarios favoring interorganellar transfer have been proposed, including scenarios leading to mitochondrial membrane disruption, such as excessive production of reactive oxygen species, release of cytochrome c, and mitophagy, the latter being impaired in senescent cells. As reviewed by Kumar and Reichert (2021), dysfunctional mitochondria are subjected to different quality control mechanisms, with different pathways operating at different extents of mitochondrial damage. Complete mitochondria are removed by mitophagy in response to severe mitochondrial damage or failure of other quality control mechanisms. Removal of mitochondria is known to negatively affect the development of many features associated with senescence, including SASP, while maintaining cell cycle arrest (Hubackova et al., 2019).

To determine whether critical components belonging to the functional units of mitochondrial clearance, mitophagy, and mitochondrial integrity have different age-associated expression profiles, we performed RNAseq of proliferative and senescent IMR-90 cells. Of the 26 genes analyzed for this purpose, three genes show significant changes in their expression, with *MSRB2* downregulated in our senescent data and *MFN2* and *BCL2L13* upregulated. We speculate that these changes in expression of genes actively involved in mitochondrial homeostasis in senescent IMR-90 cells reflect impaired mitochondrial quality control, most likely leading to cytoplasmic mtDNA accumulation in cellular senescent IMR-90 cells. According to Barja (2017), mitochondrial ROS production breaks mtDNA into mtDNA fragments that are subsequently transferred to the nucleus. It is widely believed that this extrachromosomal DNA is often used as a filler or glue in NHEJ repair of DSB. Consistent with this, Hazkani-Covo and Covo (2008), comparing pedigree-specific *numts* in humans and chimpanzees with the rhesus outgroup, uncovered sequence patterns before and after numtogenesis characterized by microhomologies and nucleotide additions reminiscent of NHEJ. Obviously, this filler strategy appears to be evolutionarily conserved. We therefore wondered whether cellular senescence drives *de novo* nuclear integration events of mtDNA in IMR-90 cells and pursued two experimental strategies, first by bioinformatic screening of WGS data and second after enrichment of nuclear DNA by an AluScan-PCR approach. Regarding the first approach, a recently published analysis on human brain and circulating immune cells together with an analysis of human fibroblasts revealed a gradual accumulation of *numts* with age (Zhou et al., 2023), using WGS data and applying bioinformatic screens for mtDNA paralogs physically linked to GRCh37 chromosomal flanks. In our datasets obtained for the two experimental approaches comparing proliferative and senescent IMR-90, we managed to identify 79 *de novo numts* of which 13 were found in the first WGS data set, 55 in the second WGS data set and 11 in the AluScan Seq data. We interpret the lower number of detectable *de novo numts* in the AluScan dataset as a result of a non-optimal representation of the Alu-intervening segments and the fact that *numts* integrate in remnants of transposable elements such as, e.g., SINEs at a frequency of 9% (see below). The latter cannot be represented by our strategy applying Alu-outward primers. In general, we corroborate data as obtained for brain regions, PBMC and fibroblasts from humans as well as data on yeast aging which is apparently influenced by the migration of mtDNA into the nucleus, too (Cheng and Ivessa, 2010). Thus, numtogenesis increases with age over a broad evolutionary divergence and is recapitulated in the IMR-90 system. To examine if there are mtDNA regions with a higher mobility rate in the process of numtogenesis, we checked which mitogenomic segments were part of our *de novo numts* sequences in our BLAT output data. Our results on mtDNA mobility and *numts* uncovered, that *de novo* integrated mtDNA paralogs consisted of segments/genes decoding MT-ND5 (10%), followed by MT-CYB (9%) and MT-RNR2, MT-ND1, MT-ND2, MT-CO1. MT-CO2 (8%). Furthermore, MT-RNR2, MT-ND1, and MT-ND2 share the same feature count in our senescent data (7.6%) and with a lower fraction of 6%, *de novo numts* apparently originate from the IMR-90 specific D-Loop. Overall, we cannot recognize a significantly preferred mobility with respect to different mtDNA regions. To elucidate a possible gene-specific impact caused by insertional mutagenesis, somatic target sites of *de novo numts* in senescent IMR-90 cells were first annotated for hg38 features applying HOMER software. We observed most target sites of senescence-associated *de novo* *numts* in introns (30%), followed by intergenic regions (24%), LINEs (19%) and SINEs (9%, Figure 8). For the latter we speculate that an open chromatin status pertaining to TEs in senescent cells potentially renders these sequences more amenable to integrations. It should be questioned if mitochondrial dysfunction is merely an epiphenomenon of senescence, or to what extent dysfunctional mitochondria, caused by insertional mutagenesis into specific genes, can possibly trigger the senescent phenotype. Several human diseases, including Pallister–Hall syndrome and mucolipidosis, can be initiated by mtDNA insertion mutagenesis of nuclear DNA (reviewed in Srinivasainagendra et al., 2017), therefore, further analyses of functional integrations patterns in conjunction with specific genes need to be performed to establish a conclusive link to cellular senescence.

Overall, we obtained evidence for mobile elements that actively cause structural genomic changes that go beyond epigenetic modifications within cellular senescent IMR-90 cells. Similar to the consequences of telomere attrition, the chance to cause larger scale rearrangements due to inequal recombination mechanisms caused by TEs is likely to be increased by TE-*de novo* integrations. Moreover, the structural variants thus generated could lead to insertional mutagenesis of relevant genes or changes in their regulation, potentially impacting processes associated with replicative aging of IMR-90 cells. Finally, our results demonstrate that non-autonomous SVA elements efficiently exploit the L1-machinery in trans in an age-dependent manner, leading to cytoplasmic cDNA derived from both SVAs, and probably other non-automomous TEs - as well as from LINE-1. De Cecco et al. (2013) stated that L1 cDNA is an important inducer of IFN-I in senescent cells, and it is reasonable to assume that this promotes an age-associated sterile inflammation, dubbed inflammaging. The same holds for cytoplasmic DNA from other sources such as mtDNA triggering IFN-1 responses which can be differentiated in both intracellular and extracellular effective pathways (West et al., 2015). Efficient counterstrategies to reduce the generation and the genomic effects of mobile cDNA and the associated innate immune response are likely candidates to therapeutically influence healthspan.

## Data Availability

The datasets presented in this study can be found in online repositories. The names of the repository/repositories and accession number(s) can be found below: https://www.ncbi.nlm.nih.gov/ PRJNA1003532.
