# Supplementary material for: Generation of somatic de novo structural variation as a hallmark of cellular senescence in human lung fibroblasts
Source: Front Cell Dev Biol. 2023 Dec 13;11:1274807. doi: 10.3389/fcell.2023.1274807 (PMC10751365; doi:10.3389/fcell.2023.1274807)
Supplement: Supplementary file 3 [file DataSheet1.docx]

Supplementary Material

Generation of somatic de novo structural variation as a hallmark of cellular senescence in human lung fibroblasts

Valentina Woronzow^1^*, Jonas Möhner^1^, Daniel Remane^1,2^, Hans Zischler^1*^

*** Correspondence:** Corresponding Author: [zischler@uni-mainz.de](mailto:zischler@uni-mainz.de), [vaworonz@uni-mainz.de](mailto:vaworonz@uni-mainz.de)

#
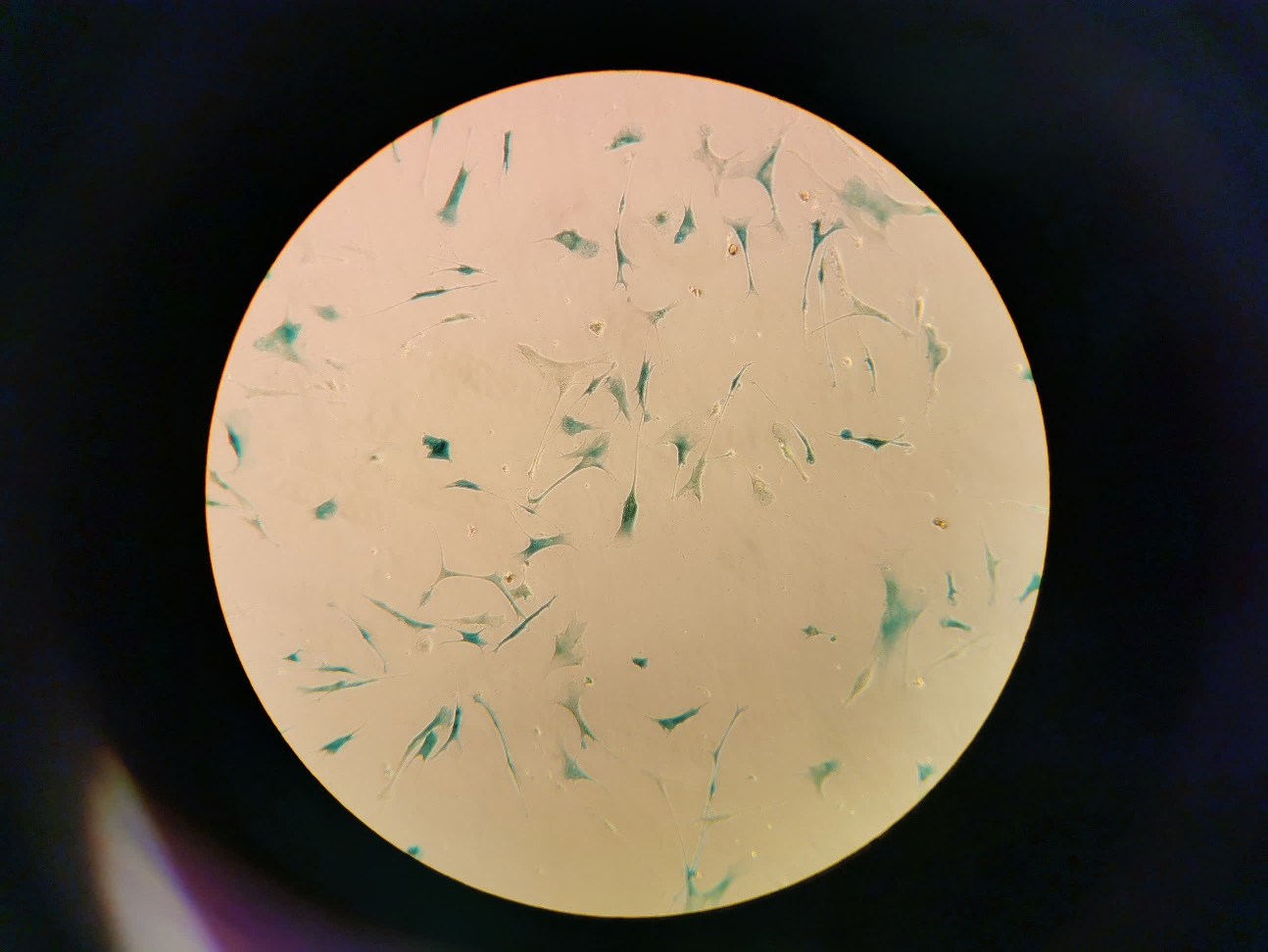

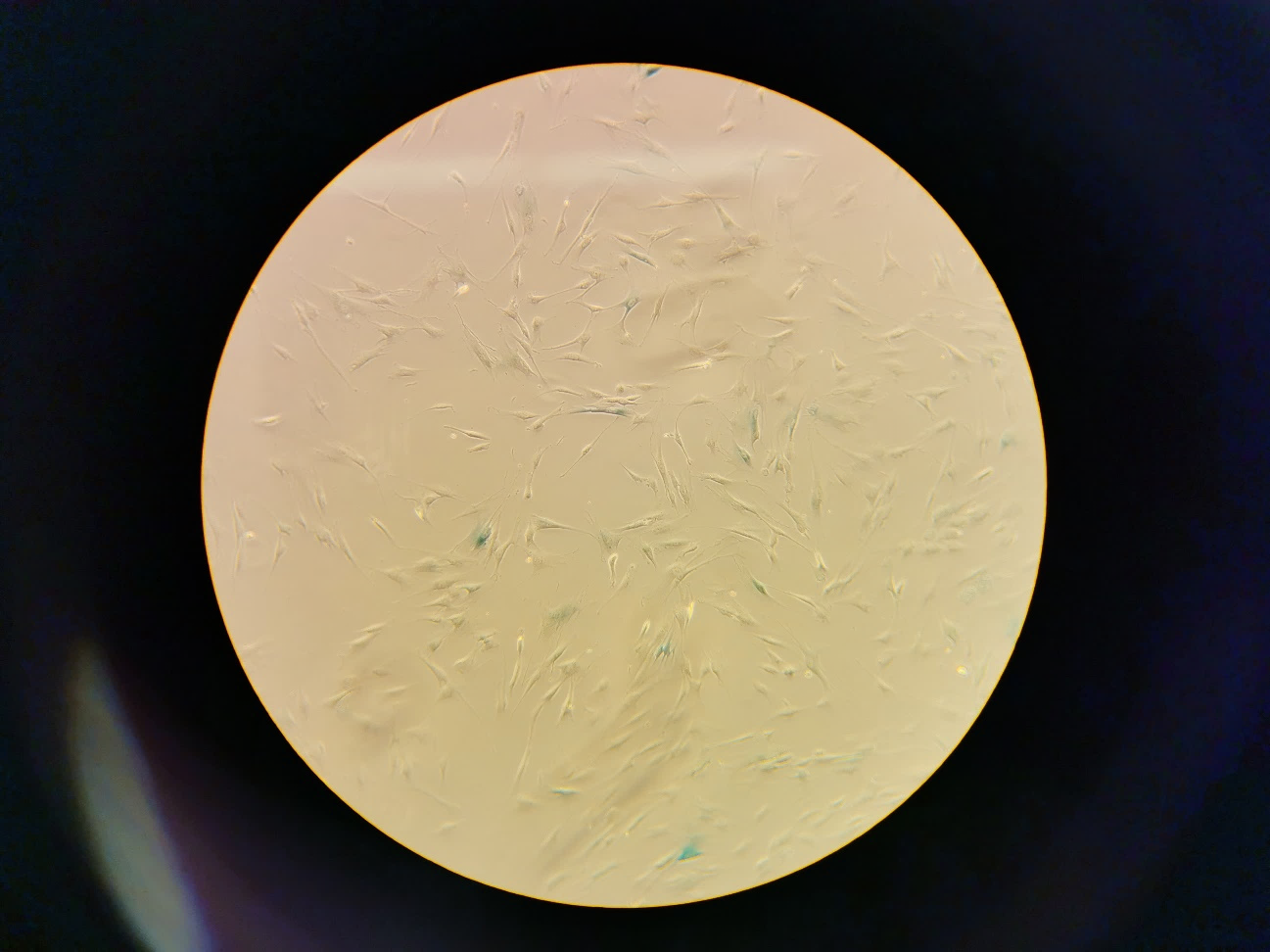
Supplementary Figures

Supplementary Figure 1 – assessment of cellular senescence in IMR-90 cells; (A) diagram depicting population doubling level (y-axis) over a time span (x-axis: time in days) of Sen_3 replicate; (B) photo documentation (100x) of negative SA-ß-Gal assay of proliferating IMR-90 cells (control); (C) photo documentation (100x) of positive SA-ß-Gal assay of senescent IMR-90 cells.

**C**

**BB**

**A**

#
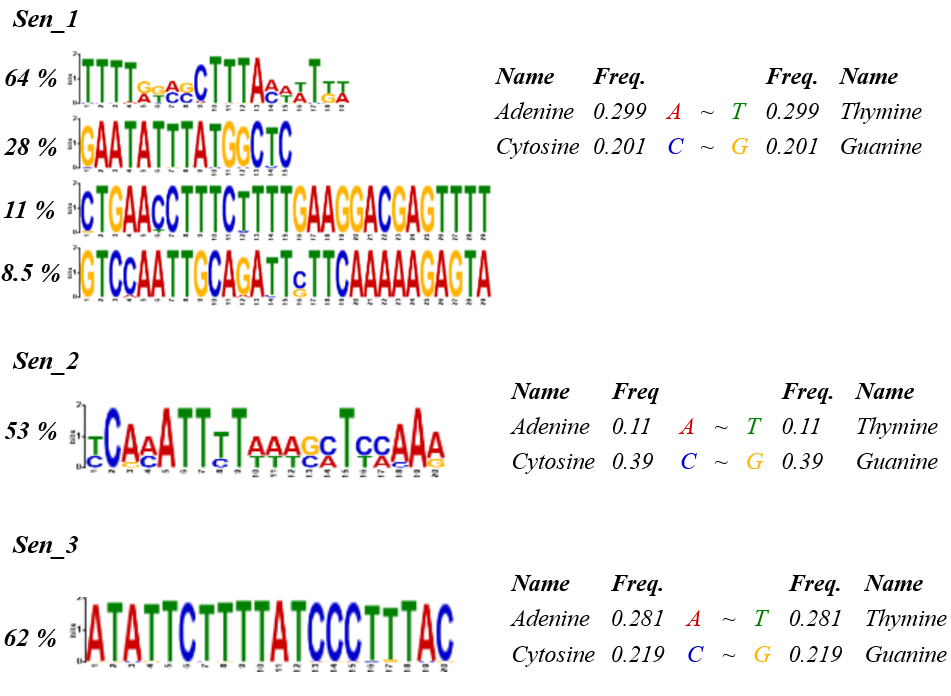


Supplementary Figure 2 – motifs associated with L1 cleavage sites identified by MEME discovery suite for the three *de novo* SVA insertion replicates (Sen_1-Sen_3). Percentage indicates quantity of identified sites within the three data sets contributing to the motifs depicted. For each sample (Sen_1-Sen_3), tables with AT and CG content of the reads associated with *de novo* SVA insertions are additionally depicted.
